# Assessing hospital antibiotic stewardship program (ASP) implementation: validation of an implementation science-informed survey

**DOI:** 10.1017/ash.2025.65

**Published:** 2025-06-20

**Authors:** Jorie Michaela Butler, Ellen Childs, Tamar Barlam, Mari-Lynn Drainoni, Caitlin Reardon, Yue Zhang, Laura Damschroder, Peter Taber, Karl Madaras-Kelly, Matthew Goetz, Shana Burrowes, Eddie Stenehjem, Jincheng Shen, Chong Zhang, Angela Presson, Matthew Howard Samore

**Affiliations:** 1 Department of Biomedical Informatics, University of Utah School of Medicine, Salt Lake City, UT, USA; 2 Division of Geriatrics, Department of Internal Medicine, University of Utah School of Medicine, Salt Lake City, UT, USA; 3 Geriatrics Research, Education, and Clinical Center (GRECC) Salt Lake City, UT, USA; 4 IDEAS Center of Innovation, VA Salt Lake City Health Care System, Salt Lake City, UT, USA; 5 Abt Associations, Rockville, MD, USA; 6 Boston University Chobanian & Avedisian School of Medicine, Boston, MA, USA; 7 Boston University School of Public Health, Boston, MA, USA; 8 VA Ann Arbor Healthcare System, Ann Arbor, MI, USA; 9 Division of Epidemiology, Spencer Fox Eccles School of Medicine, University of Utah, Salt Lake City, UT, USA; 10 Idaho State University, Meridian, ID, USA; 11 VA Greater Los Angeles Healthcare System, Los Angeles, CA and David Geffen School of Medicine at UCLA, Los Angeles, CA, USA; 12 Intermountain Healthcare, Salt Lake City, UT, USA

## Abstract

**Objective:**

Antibiotic stewardship programs (ASPs) are crucial to prevent the emergence of antibiotic resistance and to improve outcomes for patients. A validated instrument rooted in a theoretically derived implementation science framework will increase our understanding of ASP implementation and enable comparisons across implementation sites.

**Design:**

**Methods:**

Antibiotic stewards (infectious disease pharmacists and physicians) were recruited from Veterans Affairs (VA) hospitals to complete a survey on stewardship implementation. We used the Consolidated Framework for Implementation Research (CFIR) to guide development of an ASP implementation survey assessing 22 potential determinants of implementation across five domains of CFIR. We conducted confirmatory factor analyses (CFA) to assess construct validity of 8 construct measures and evaluated internal consistency.

**Results:**

A total of 150 stewards completed the survey from 110 VA hospitals. CFA for most CFIR constructs exhibited good fit. Internal consistency for CFIR construct subscales (Cronbach’s alpha) ranged from 0.54–0.96, indicating modest to strong internal consistency. Determinants that were rated highly present at the sites (across site means ≥ 4.0 or above) included Self-Efficacy, Engaging, Evidence Strength and Quality and Relative Advantage, indicating stewards found ASP evidence compelling and felt their personal involvement was effective in engendering positive results for the ASP.

**Conclusions:**

Psychometric properties indicate validity of the first CFIR-based survey of determinants for ASP implementation outcomes. Clinical, quality improvement, and research teams can use this survey to identify contextual determinants of ASP implementation and use this information to guide selection of strategies and compare results across multiple sites.

## Background

Antibiotic stewardship programs (ASPs) are successful in reducing inappropriate prescribing, improving patient outcomes, and curbing antibiotic resistance and are now required for hospitals by the Centers for Medicare and Medicaid Services.^1^ ASP implementation may include a wide range of activities such as prospective audit and feedback, de-escalation, educating clinicians, tracking antibiotic use patterns, and reporting to leadership and government agencies.^1^ ASPs involve complex interventions with multiple components including activities to support both individual patient health and population health, and effective communication with physicians and staff. Implementation of ASPs across diverse hospital settings provides crucial opportunities to compare experiences and also to identify determinants of successful ASP implementation.

Despite widespread recognition of the importance of ASPs, few accepted surveys exist to assess their implementation grounded in direct feedback from antibiotic stewards. Existing surveys of stewards have focused on other factors relating to antibiotic stewardship practice but have not specifically examined the implementation process.^2,3^ While some ASP surveys have addressed specific implementation activities, none have used a theoretically derived implementation science determinant framework to identify facets of ASP implementation that may differentiate between more and less successful programs. To address the lack of validated surveys that assess implementation processes and identify determinants of successful ASP implementation, we developed a survey for antibiotic stewards using the Consolidated Framework for Implementation Research (CFIR).

Implementation is a complex endeavor characterized by social and contextual facets.^4–7^ The CFIR is rooted in knowledge from many disciplines, including organizational change and psychology. CFIR provides a conceptual foundation for studying implementation by defining a “menu” of constructs potentially associated with implementation effectiveness and providing a systematic, comprehensive, and tailorable approach to uncovering drivers of variability in implementation outcomes prospectively. The CFIR is useful for determining pathways to sustained intervention success as each construct represents a theoretically-based determinant.^8^ Psychometric validation incorporates methods to assess measurement properties to determine whether a measure is assessing what it intends to measure. Psychometric validation of CFIR consistent survey measures has been used to identify optimal measures of implementation for pediatric Intensive Care Units and in behavioral health.^9,10^

The CFIR consists of five broad domains: 1. ***Characteristics of the Intervention***, 2. ***Outer Setting***, 3. ***Inner Setting***, 4. ***Characteristics of Individuals***, and 5. ***Process***. Domains are comprised of constructs (39 in total) that describe more specific components of the domain. The CFIR has been used to assess implementation of many types of innovations across diverse settings.^11–13^ Some constructs have not been quantitatively measured, limiting the opportunity for survey validation, and others have been measured only rarely. In 2016, Clinton-McHarg and colleagues conducted a review of survey measures aligned with CFIR constructs and administered in public health and community settings^14^ and found that 5 of the 39 CFIR constructs were not included by any of the measures evaluated. Our objective was to develop survey measures of determinants of implementation success across the CFIR’s 5 domains and assess the psychometric validity of those measures in the context of ASP implementation. This validation will permit future work examining implementation of ASPs across facilities using these survey measures.

## Methods

*Study Settings and Approach:* The current study is one component of a larger mixed-methods study of antibiotic stewardship at 20 Intermountain Healthcare hospitals and 134 Veterans Health Administration (VHA) Medical Centers across the United States.^15^ Our study examines the psychometric properties of the CFIR-based survey of ASP stewards in VHA settings only. We evaluated the factor structure of the survey using confirmatory factor analysis (CFA), the appropriate technique when there is a theoretical foundation underlying the expectations for the data structure.^16,17^ CFA is designed to identify latent constructs in a data structure. Latent constructs are not directly observable but can be inferred from survey items. General examples include a construct such as motivation. Motivation cannot be measured directly but could be inferred based on specific questions assessing interest in performing a task.

Implementation science concepts – such as engaging – are latent constructs. Assessing whether the data structure based on survey items is, in practice, consistent with expected latent constructs in alignment with theory provides evidence for validity of the survey. We also assessed face validity (whether the questions seem to represent the constructs), discriminant validity of the constructs (statistical evidence that the constructs were measuring the distinct concepts), and internal consistency of the items within each construct (indicating that the items align with each other in measuring a similar construct). We reviewed site-level survey data to assess potential determinants (barriers and facilitators) to antibiotic stewardship implementation that can later be tied to implementation outcomes.

*Survey Development and Characteristics:* The study team developed initial survey items via team collaboration. Members of the study team who are antibiotic stewardship experts (MS, TB, MG, KMK, ES) and survey methodology, implementation science, or CFIR-specific experts (CR, LD, MLD, JB) used the online CFIR technical assistance website^18^ to develop survey items consistent with antibiotic stewardship implementation in CFIR-recommended structure to assess constructs. Candidate questions were discussed by the entire study team at length and reviewed by key CFIR experts (LD, CR) and the modified survey was piloted with antibiotic stewards. Final revisions incorporated suggestions from all levels of review and piloting.

The administered survey comprised 72 items representing all five CFIR domains and 22 CFIR constructs considered relevant to antibiotic stewardship implementation. Survey items were rated on a Likert scale from 1 to 5, where 1 represented “strongly disagree” 2 “disagree, 3 “neither agree nor disagree” 4 “agree” and 5 represented “strongly agree,” with an additional “don’t know” option (survey items in results; Table 1).

**Table 1.** Survey descriptive summary

Domain, Constructs, and items (variable name).					
**Domain: Inner Setting**	Mean (SD)	Floor%	Ceiling%	UnK%	#Miss
**Construct 1: Culture**	3.7 (0.64)	1%	3.80%		6
Staff at the hospital have a sense of personal responsibility for improving patient care and outcomes (personal)	3.8 (0.7)	0.90%	8.30%	1.90%	2
Staff at the hospital cooperate to maintain and improve effectiveness of patient care (maintain_effectiveness)	3.9 (0.7)	0.90%	9.20%	2.80%	1
Staff at the hospital are receptive to change in clinical processes (receptive_to_change)	3.4 (0.8)	2.80%	3.70%	3.70%	1
**Construct 2: Readiness for implementation: Leadership Engagement**	3.4 (.65)	0%	0%		10
*Our hospital primarily started the ASP because it was mandated	3 (1.3)	13.60%	15.50%	0.90%	0
There was an internal push from hospital leadership to establish an ASP program (intervention_source_2)	2.9 (1.2)	10%	5.50%	7.30%	0
There was an ASP champion on the clinical staff who actively promoted the implementation of the ASP (champion)	4.1 (1)	1.80%	35.50%	3.60%	0
Clinical leadership has endorsed the ASP in visible ways (transparent)	3.7 (1)	4.50%	13.60%	0.90%	0
Clinical leadership gives the antibiotic steward the authority to enforce the ASP policies (autonomy)	3.8 (0.9)	1.80%	14.70%	0.90%	1
The Antibiotic steward has protected time to implement the ASP (protected_time)	3.1 (1.2)	9.10%	10.90%	0%	0
**Construct 3: Readiness for implementation: Access to Knowledge**	3.2 (.80)	1%	1%		5
The EHR provides helpful reports about antibiotic use in the hospital (investment)	3 (1.1)	9.30%	7.40%	0.90%	2
Clinicians and hospital staff have received enough education and training on the ASP (enough_training)	3.2 (1)	3.60%	6.40%	0%	0
The ASP is integrated into new provider training (new_provider_training)	3.5 (1)	2.70%	13.60%	2.70%	0
**Domain: Characteristics of Individuals**					
**Construct 4: Knowledge and Beliefs about Intervention**	3.7 (.45)	0	0		14
The staff was receptive to the ASP (receptive)	3.8 (0.7)	0%	11.80%	0.90%	0
Nursing and other support staff understand the importance of the ASP policies (understand_importance)	3.5 (0.8)	0.90%	7.40%	4.60%	2
*ASP policies put a heavy burden on the nursing staff (nursing_burden)	4 (0.7)	0.90%	13.60%	1.80%	0
Clinical Pharmacists understand the importance of the ASP policies (pharmacist_importance)	4.4 (0.7)	0%	45.90%	0.90%	1
*ASP policies put a heavy burden on Clinical Pharmacists (pharmacy_burden)	3 (1)	8.20%	2.70%	3.60%	0
*Clinicians do not like the ASP because they feel it limits their ability to treat patients the way they think is best (limiting)	3.5 (0.9)	1.80%	7.30%	6.40%	0
*Clinicians think the ASP delays antibiotic therapy too much (delay)	3.8 (0.7)	0.90%	8.20%	6.40%	0
*Clinicians think the ASP restricts too many antibiotics (restricting)	3.5 (0.9)	0.90%	6.40%	4.50%	0
*The ASP involves too many steps for clinicians to adhere to in prescribing antibiotics (excessive)	3.9 (0.7)	0%	11.90%	1.80%	1
**Construct 5: Self-Efficacy**	4.1 (0.56)	0%	4.7%		4
I have a lot of authority in the antibiotic decisions made at my facility (authority)	3.9 (0.8)	0.90%	17.40%	0%	1
I offer clinicians options regarding antibiotic decision making at my facility (decision making)	4.3 (0.5)	0%	23.60%	0%	0
I accept responsibility for the outcomes of this program (responsible_for_outcomes)	4.1 (0.7)	0.90%	21.10%	0%	1
I feel like I can effect change at my facility (hopeful)	4 (0.8)	2.80%	16.70%	0%	2
I feel like I have the skills to function effectively in my role (skillful)	4.1 (0.7)	1.80%	20%	0%	0
I am able to carry out the mission of the ASP at my hospital (accomplishment)	4 (0.8)	1.80%	19.10%	0%	0
I am empowered to continue to improve the ASP (empowered_to_improve)	4.1 (0.8)	0.90%	22.70%	0%	0
I am invested in the success of the ASP (invested_in_success)	4.4 (0.6)	0%	35.80%	0%	1
**Domain: Process**					
**Construct 6. Planning/Execution**	3.6 (0.58)	0%	0%		11
When the ASP was initially developed roles and responsibilities were clearly identified (roles)	3.4 (1)	3.60%	7.30%	1.80%	0
A realistic time schedule was developed for implementation of the ASP when the program was initially implemented (timeframe)	3.6 (0.9)	3.60%	7.30%	8.20%	0
The original plan for implementing the ASP acknowledged clinicians input and opinions (input)	3.8 (0.8)	1.80%	12.70%	3.60%	0
*The original plan for implementing the ASP was unnecessarily complex (complex)	3.7 (0.8)	1.80%	10%	1.80%	0
Nurses are actively engaged in the ASP activities (nurses_engagement)	2.7 (0.8)	1.80%	0.90%	0.90%	0
Clinical Pharmacists are actively engaged in ASP activities (pharmacy_engagement)	4.2 (0.8)	1.80%	34.50%	0.90%	0
**Construct 7. Engaging**	4.0 (0.53)	0%	2.8%		4
The ASP had the support of the key opinion leaders in the hospital (support)	3.6 (1)	4.50%	11.80%	2.70%	0
The ASP has considerable visibility within the hospital (visibility)	3.7 (0.9)	2.80%	13.80%	0%	1
I work well with the interdisciplinary medical teams (collaborative)	4.3 (0.5)	0%	29.10%	0%	0
I work well with individual clinicians (individual)	4.4 (0.5)	0%	28.20%	0%	0
**Construct 8. Reflecting and Evaluating**	3.5 (.56)	0%	0%		14
Hospital leadership receives regular feedback on progress of ASP activities and resource needs (feedback_on_progress)	3.8 (0.9)	0.90%	17.30%	1.80%	0
Feedback from clinicians related to proposed and implemented changes of the ASP is collected regularly (feedback_changes)	3 (1)	4.60%	5.60%	2.80%	2
Clinical leadership at the hospital provides staff with information on hospital performance measures and guidelines (aware_of_measures)	3.8 (0.7)	0.90%	9.20%	2.80%	1
Clinical leadership at the hospital establishes clear goals for patient care processes and outcomes (clear_goals)	3.6 (0.8)	0.90%	6.40%	4.60%	1
Clinical leadership at the hospital provides staff with feedback data on effects of clinical decisions (data_on_effects)	3.3 (0.8)	0.90%	2.80%	5.50%	1
Clinical leadership at the hospital hold staff accountable for achieving results (accountable)	3.5 (0.8)	0.90%	6.50%	6.50%	2

Note. * indicate reverse-coded items.

*Recruitment and Participants*: We identified 289 physician and pharmacist antibiotic stewards at VHA hospital sites based on a list of persons in those roles reported through VA surveys, identification of role on websites, or partners. In January 2018, we sent emails to each VA steward inviting them to complete the REDCap survey online.^19^ At least one response was obtained from each of 110 VHA hospitals.^20^ At the hospital level, the response rate was 81% whereas the individual steward response rate was 52%. Our analysis was at the hospital level, and 81% is a high response rate. A comparison of emographics respondents and non-respondents demonstrated significant differences in role of respondent between groups (Table 2).

### Psychometrics evaluation of the antibiotic steward CFIR survey:

Although our survey measures assessed all 5 CFIR domains, we evaluated the psychometric properties of the 3 CFIR domains and 8 survey measures of constructs with three or more items. The methods used to evaluate the psychometric properties of the construct using CFA require a minimum of 3 items. For transparency and to support other research, all survey questions are included in Table 1. For hospitals with more than one survey respondent, responses were aggregated at the hospital level by averaging them. In the final analysis, 110 hospitals were included, among which 40 had more than 1 respondent. We reverse-scored items that were measuring the trait in the opposite direction (see asterisks by items in Table 1). All analyses were done at the hospital level.

We assessed the internal consistency and the unidimensional contribution of each construct using Cronbach’s alpha and McDonald’s Omega. Omega uses a more conservative standard with purportedly less bias, thus we present both.^21^ Internal consistency, an indicator that a group of questions are measuring the same underlying concept, was considered acceptable if >0.7.^22,23^ Floor and ceiling rates were provided for each construct and individual items to demonstrate the percent of time respondents chose the lowest possible (floor) or highest possible (ceiling) rating for each item (or construct). For constructs, we considered floor and ceiling rates at <10% to be acceptable. To assess discriminant validity, we examined correlations between constructs. Correlations below 0.80 are considered below threshold and indicate good discriminant validity. Correlations above 0.80 suggest measurement overlap between constructs.^24^

We performed CFAs to assess whether the expected theoretical CFIR construct from our survey on antibiotic stewardship implementation was supported by the survey data. We used the LAVAAN statistical package available in R for analyses.^25^

For constructs with ≥4 items, we fitted single-factor congeneric models. Constructs with only 3 items result in saturated congeneric models, which cannot be evaluated for goodness-of-fit. In such cases, we used the more restrictive tau-equivalent model, which assumes that the item loadings are equal. Item loadings represent a correlation between specific items (eg, survey questions) and the underlying factor. Thus in tau-equivalent models, each item is constrained to contribute equally to the factor. For congeneric models with inadequate fit, we relaxed the assumption that item residuals were uncorrelated. Modifying models to allow for correlated item residuals is appropriate when justified both statistically and from theoretical models of the items.^26^ In leadership engagement, items relating to drivers of the intervention (eg, mandates), authority, and structure (protected time) were allowed to have correlated residuals. In *Knowledge and Beliefs about Intervention* items related to receptivity to the intervention (receptivity and understanding) as well as concerns (limits on autonomy and delays) were allowed to have correlated residuals. For the construct *Engaging*, items related to perceived success in collaboration (with teams or other individuals) were allowed to correlate. For transparency, the fit indices for congeneric models without the relaxed assumption are available in supplementary materials.

To assess model fit, we used widely recommended indices.^27–29^ In these models we assessed Chi square (non-significant value = good fit), comparative fit index (CFI>0.95 = good fit), the Tucker-Lewis index (TLI>0.95 = good fit), the root mean square error of approximation (RMSEA, <0.08 = good fit) and standardized root mean square residual (SRMR<0.05 = good fit, <.08 = mediocre fit).

**Figure 1.**
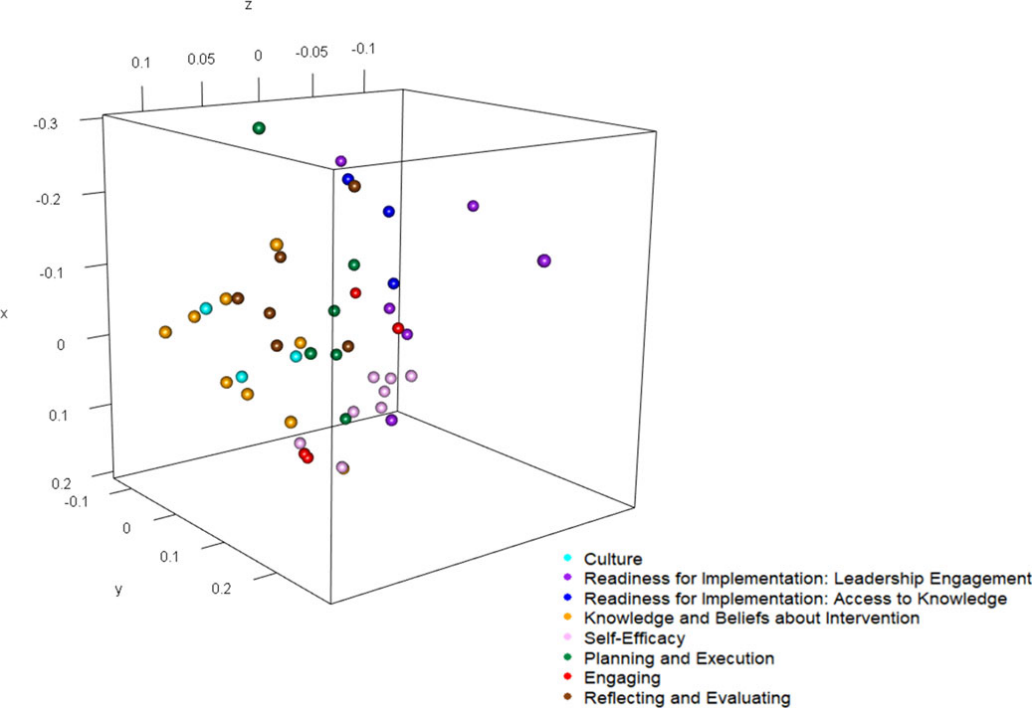
Relationships among CFIR items by Construct.

We used a multidimensional scaling plot to visualize relationships among items and constructs. Since there was a mixture of Likert and continuous items, relationships were quantified using Gower’s distance.^30^ We used uniform coloring for items within a construct (see Figure 1).

**Figure 2.**
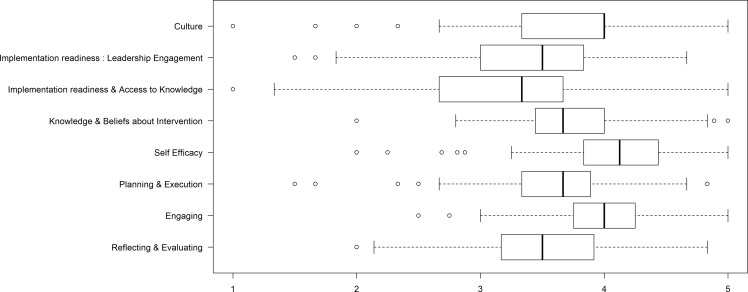
Construct responses.

**Figure 3.**
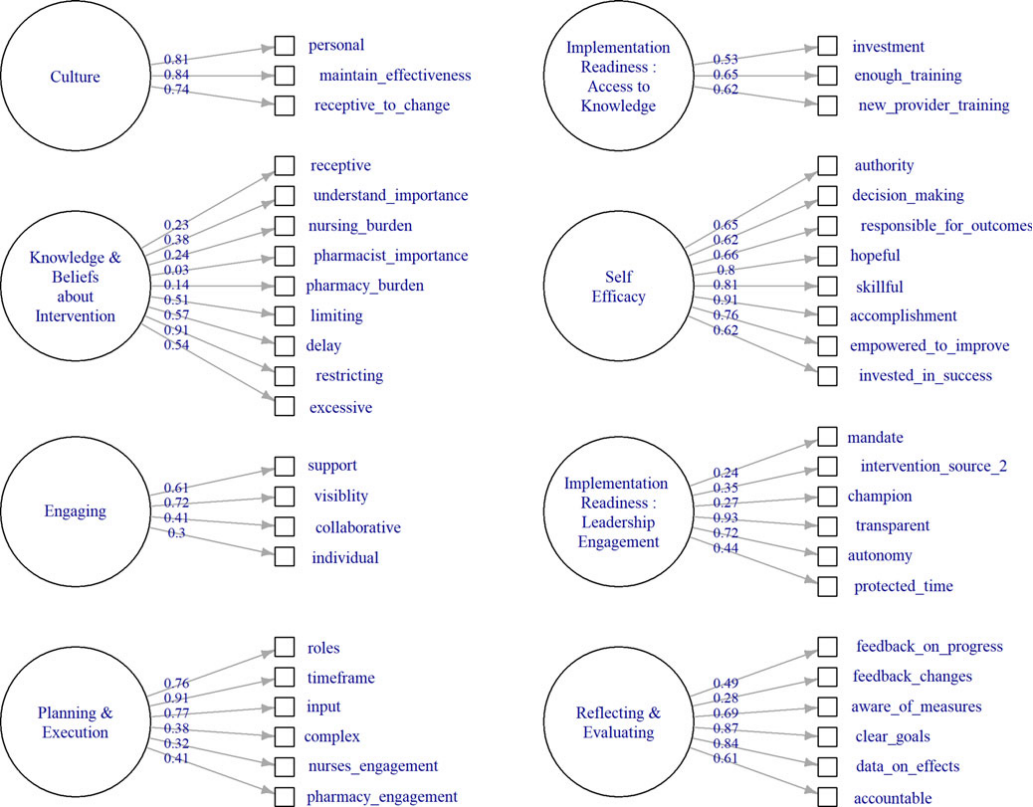
Factor loadings by CFIR construct.

## Results

A total of 110 hospitals participated in this survey.^20^ Survey item mean scores ranged from 3.0–3.9 for 28 of 43 items (65%) indicating that the average response at the hospital level for those items was between “neither agree nor disagree” and “agree.” (Table 1). Construct means ranged between 3.2 (*Access to Knowledge*) and 4.0 (*Knowledge and Beliefs About the Intervention*) (Figure 2). High-ranked individual items exhibited ceiling effects. Ceiling effects were most pronounced within the construct *Readiness for Implementation: Leadership engagement*. Internal consistency was acceptable to high for 6 of the 8 constructs and marginal (>0.70) for the remaining 2, construct 6, *Access to Knowledge* and construct 7, *Engaging*, which had alpha values of 0.66 and 0.61, respectively.

*Model Fit.* The fit of models was excellent for 4 models –representing two constructs in the ***Inner Setting*** domain; *Culture* and *Leadership Engagement* and for two constructs in the ***Process*** domain *Planning/Executing*, and *Engaging*. (Table 3). For 3 models, the fit was adequate, one in ***Inner Setting***, construct *Access to Knowledge and Information*, one in ***Characteristics of Individuals***, construct *Knowledge and Beliefs About the Intervention*, and one in ***Process*** domain, *Reflecting and Evaluating*. The model in ***Characteristics of Individuals***, construct *Self-efficacy* had mediocre fit but high reliability.

**Table 2.** Comparison of respondents and nonrespondents

	Respondents (N = 155)	Nonrespondents (N = 134)	P value
**Gender**			
Female	83 (53.5%)	68 (50.7%)	0.721
Male	72 (46.5%)	66 (49.3%)	
**Region**			
Continental	29 (18.7%)	24 (17.9%)	0.408
Northeast	56 (36.1%)	58 (43.3%)	
Pacific	27 (17.4%)	15 (11.2%)	
Southeast	41 (26.5%)	37 (27.6%)	
Missing	2 (1.3%)	0 (0%)	
**Role**			
Pharmacist	101 (65.2%)	55 (41%)	*<*0.001
Physician	53 (34.2%)	79 (59%)	
Missing	1 (0.6%)	0 (0%)	

**Table 3.** Construct mean, models and internal consistency (α,θ) and indications of fit

CFIR Construct	Mean (SD)	Floor	Ceiling	Alpha	Omega	Chisq	df	p value	CFI	TLI	RMSEA	SRMR	N
Culture*	11.1 (1.9)	1%	3.8%	0.84	0.84	0.55	2	0.76	1	1.02	0	0.03	104
Readiness for implementation: Leadership Engagement^	20.5 (4.0)	0	0	0.66	0.69	6.45	6	0.37	1	0.99	0.03	0.03	100
Readiness for implementation: Access to Knowledge*	9.7 (2.4)	1%	1%	0.61	0.63	2.06	2	0.36	1	1	0.02	0.06	105
Knowledge and Beliefs about Intervention^	33.6 (3.7)	0	0	0.69	0.7	35.75	23	0.04	0.93	0.89	0.08	0.07	96
Self-Efficacy	32.9 (4.5)	0	4.7%	0.9	0.9	50	20	<0.001	0.94	0.91	0.12	0.05	106
Planning/Execution	21.4 (3.5)	0	0	0.76	0.78	8.1	9	0.52	1	1.01	0	0.04	99
Engaging^	16.0 (2.1)	0	2.8%	0.69	0.73	0.09	1	0.76	1	1.04	0	0	106
Reflecting and Evaluating	21.0 (3.3)	0	0	0.8	0.81	11.9	9	0.22	0.98	0.97	0.06	0.05	96

*Use tau equivalent model – constraining loadings to be equal.

^Allowing correlations among residuals.

Factor loadings were consistently high for construct 5, *Self Efficacy.* For construct 4, *Knowledge and Beliefs about the Intervention* (see Figure 3 low loadings were found on items relating to overall staff receptivity, nursing burden, pharmacy burden, and pharmacist ratings of importance For construct 7, *Engaging*, the item “I work well with individual clinicians” loaded poorly. High factor loadings represent a strong relationship between the individual item and the latent factor whereas low loadings suggest complexity in the relationship between these items and the factor.

*Discriminant validity:* Pairwise correlations between constructs were below the threshold of 0.80, indicating acceptable discriminant validity, with one exception. Construct 5, *Self-Efficacy*, and Construct 6, *Engaging*, had a correlation coefficient of 0.81, thus failing the test for discriminant validity (see Table 4).

**Table 4.** Correlations between constructs

CFIR Construct	Culture	Readiness for Implementation: Leadership Engagement	Readiness for Implementation: Access to Knowledge	Knowledge and Beliefs about the Intervention	Self-Efficacy	Planning and Execution	Engaging	Reflecting and Evaluation
Culture*	1	.37	.44	0.26	.39	.35	.35	.53
Readiness for implementation: Leadership Engagement^	.37	1	.50	.44	.66	.70	.67	0.58
Readiness for implementation: Access to Knowledge*	.44	.50	1	.27	.49	.53	.57	.61
Knowledge and Beliefs about Intervention^	.26	.44	.27	1	.31	.44	.43	.31
Self-Efficacy	.39	.66	.49	.31	1	.60	.81	.53
Planning/Execution	.35	.70	.53	.44	.60	1	.61	.60
Engaging^	.35	.67	.57	.43	.81	.61	1	.54
Reflecting and Evaluating	.53	.58	.61	.31	.53	.60	.54	1

*Multidimensional Scaling:* We screened correlations between individual items of the two construct scales which had below acceptable discriminant validity to understand the relationship between items. The highest correlation was 0.66 which was between the *Engaging* item “I work well with interdisciplinary teams” and the *Self-efficacy* item, “I offer clinicians options regarding antibiotic decision making at my facility.” Lower inter-item correlations were found for other single-scale items. The construct survey measures were highly correlated, and the specific inter-item correlations suggest similarity between the theoretical constructs (see supplement for full table). The multidimensional scaling plot indicated that items within the *Self-Efficacy* construct also particularly correlated (see Figure 1).

## Discussion

The goal of this analysis was to develop and psychometrically evaluate a CFIR-based survey instrument in the context of ASP implementation. A psychometric validation process for a survey is designed to evaluate whether a survey is measuring concepts reliably (in a consistent way) and validly (measuring the constructs it intends to measure) and is a key step in conducting research and quality improvement. We assessed the responses to the CFIR survey to determine whether the survey questions within the models met our expectations for structure and consistency and whether the individual models were independent of each other (ie, not highly correlated) and thus able to provide novel information.

Other surveys have described the development and components of antibiotic stewardship programs, but many have focused primarily on establishing stewardship across sites for comparison^31^ and exploring attitudes toward stewardship.^32^ Existing studies have demonstrated the validity of survey instruments intended to measure the ***Inner Setting*** domain and its component constructs,^33,34^ it is important to note that our study is the first to confirm construct validity in a measure in the context of antibiotic stewardship using 3 out of 5 CFIR domains. Our survey includes questions within the rarely measured ***Process*** domain, which envelops the *Champions* construct applied to stewardship.^14^ Validated survey measures that include multiple CFIR constructs advance the field of implementation science and of ASP implementation in particular.

Our survey demonstrated multidimensional validity based on our theory-based survey measure and the results of our CFA. Most of our CFA models exhibited excellent or very good fit to the data. We also demonstrated internal consistency of the survey measures. Our results showed discriminant validity for most constructs – indicating that each construct is different from the other survey constructs. Where discriminant validity was marginal, between the constructs *Engaging* and *Self-Efficacy*, there are distinct similarities between the theoretical and behavioral concepts being measured. Namely, *Engaging* addresses visibility, support, and capabilities whereas *Self-Efficacy* addresses beliefs that one can capably perform specific were actions. These similarities suggest further work. There were negligible floor and ceiling effects at the construct level. As a result, our survey should perform well at discriminating among sites with both low and high performance although this will need confirmation in future studies. Overall, our survey demonstrates psychometric validity and can be used as designed. Although we did not assess ASP implementation outcomes in this paper, validating these survey measures will allow our team and other teams to assess the relationship between these validated survey measures and implementation of stewardship programs and stewardship outcomes in future work.^35^

construct.

However, our results also bring to light some key issues that should be considered by research teams examining determinants of antibiotic stewardship implementation. First, our work points to particular determinants that may be important to better understand or measure over time as possible harbingers of ASP success. Items in the *Self-efficacy* and *Engaging* constructs related particularly to individual characteristics, with most items beginning with “I” (11 of the 13 items across the survey measures). It will be important to investigate the relationship between individual sense of agency and ASP success in future work.

In some cases, models with correlated item variance may indicate that unmeasured variables remain. For example, the *Readiness for Implementation*: *Leadership Engagement* model, we potentially identified evidence of an unmeasured variable representing the perception of a “compulsory” component of the ASP intervention demonstrated by low factor loadings for the item relating to external mandates for ASP. It is possible that stewards associate a mandate with external pressure on leadership and that this may be different from other aspects of leadership engagement. These findings point to the complex interplay between individual beliefs, autonomy and motivation, and how to identify individual versus collective forces for change. This seems particularly suitable for antibiotic stewardship environments, which must carefully weigh individual versus collective priorities and motivations.^36^

Our findings also point to *Leadership Engagement* as potentially more motivating than a mandate, which implies low autonomy. Better understanding of the interplay between a mandate and engagement could support efficient design of mature stewardship programs. Our results also suggest a potentially unmeasured construct relating pharmacist beliefs about importance of ASP interventions to general receptivity to intervention among the staff. Potentially pharmacist communication about beliefs (even non-verbal) may have an outsized influence on their colleagues. If confirmed, this finding could promote additional practical and theoretical contributions to ASP development.^37,38^ Our work points to the importance of individual cognition, motivation, and social cognitive approaches. This is consistent with other work addressing individual cognition and social dynamics for ASP interventions.^5,39^

Healthcare environments are complex, rapid-paced, cognitively challenging environments. Sociotechnical systems models and methods are designed to elucidate and solve important challenges related to communication, human-computer interaction, cognition, and motivation.^40–42^ It is imperative that we continue to tackle complex problems with the deeper, interdisciplinary approaches that sociotechnical systems and implementation science are advancing in antibiotic stewardship.

## Implications for future ASP research

This psychometrically validated survey can be used by antibiotic stewards, quality improvement staff, and researchers to assess and report ASP implementation within and/or across ASPs. This survey may be useful for exploratory assessment of implementation domains and constructs at a specific site, for example, for a prospective hospital site champion to assess leadership readiness prior to ASP implementation. It is important to note that our survey is validated for antibiotic stewards, and further work is needed to validate performance by reporters in different clinical roles.

*Limitations:* Our results should be understood in the context of the following limitations. First, we have a relatively small sample for a confirmatory factor analysis based on the hospital-level analysis, but it is important to note that for all 8 constructs we tested single-factor models, thus power should be adequate. Conducting analyses at the hospital level may also have influenced some of the domains and constructs, particularly those within the ***Characteristics of Individuals*** domain. A site-level rating of *Self-Efficacy* may represent a combined “self” across respondents when answers by multiple individuals at a single site were different. Yet, despite these limitations, the data were a good fit for the models. Our sample of intermountain health hospitals was too small to assess whether the construct validity for our survey was equally strong across different health systems. Further validation work with larger non-VHA systems will allow this type of comparison. In addition, there may be other concepts that need exploration, such as whether the “*Readiness for Implementation: Leadership Engagement*” reports could be related to social desirability influencing responses.^43^ In addition, CFIR constructs are evolving. Not all constructs included in our survey may be applicable to survey users who would like to focus on the updated CFIR.^35,44^ Finally, this work was conducted before many hospitals were challenged in 2020 and 2021 by the COVID-19 pandemic. Establishing a baseline is very important but future work may be needed to understand subsequent changes in VHA.

*Conclusions:* Validated surveys are needed to assess the implementation of antibiotic stewardship across sites. In contrast to earlier work, this robust suite of CFIR survey questions specific to antibiotic stewardship can be used to complement other data collection methods addressing stewardship implementation and promote implementation science growth. In effect, use of our survey can address the contextual components of implementation in greater detail and its relationship to ASP outcomes than has previously been possible. Our findings may guide future scale modifications for teams interested in studying ASP implementation.^21^

## Supporting information

10.1017/ash.2025.65.sm001 Butler et al. supplementary material Butler et al. supplementary material

## Data Availability

The datasets generated and/or analyzed during the current study are not publicly available due to data use restrictions but are available from the corresponding author on reasonable request with Institutional Review Board approval and a Data Use Agreement.

## Supplementary material

The supplementary material for this article can be found at https://doi.org/10.1017/ash.2025.65

## List of Abbreviations:

Antibiotic Stewardship Programs (ASPs)

Confirmatory Factor Analysis (CFA)

Comparative fit index (CFI)

Consolidated Framework for Implementation Research (CFIR)

Root mean square error of approximation (RMSEA)

Standardized root mean square residual (SRMR)

Tucker-Lewis index (TLI)

Veterans Affairs (VA)
