# Supplementary material for: Assessing hospital antibiotic stewardship program (ASP) implementation: validation of an implementation science-informed survey
Source: Antimicrob Steward Healthc Epidemiol. 2025 Jun 20;5(1):e133. doi: 10.1017/ash.2025.65 (PMC12188278; doi:10.1017/ash.2025.65)
Supplement: Butler et al. supplementary material [file S2732494X25000658sup001.rtf]

Supplementary Table. Results of Unadjusted Models subsequently adjusted to allow correlated residuals
CFIR Construct	Chisq	df	p value	CFI	TLI	RMSEA	SRMR	N	
Readiness for implementation: Leadership Engagement	37.8	9	<0.0001	.80	0.66	0.18	0.11	100	
Knowledge and Beliefs about Intervention	103.4	27	<0.001	0.58	0.44	0.17	0.12	96	
Engaging	18	2	<0.001	.88	0.63	0.28	0.11	106	


Supplementary Table – Items for which residuals were allowed to correlate
Construct 2: Readiness for implementation: Leadership Engagement 	
*Our hospital primarily started the ASP because it was mandated>+	
There was an internal push from hospital leadership to establish an ASP program (intervention_source_2)>	
There was an ASP champion on the clinical staff who actively promoted the implementation of the ASP (champion)+	
Clinical leadership has endorsed the ASP in visible ways (transparent)	
Clinical leadership gives the antibiotic steward the authority to enforce the ASP policies (autonomy)^	
The Antibiotic steward  has protected time to implement the ASP (protected_time)^	
Construct 4: Knowledge and Beliefs about Intervention 	
The staff was receptive to the ASP (receptive)>^	
Nursing and other support staff understand the importance of the ASP policies (understand_importance)>	
*ASP policies put a heavy burden on the nursing staff (nursing_burden)	
Clinical Pharmacists understand the importance of the ASP policies (pharmacist_importance)^~	
*ASP policies put a heavy burden on Clinical Pharmacists (pharmacy_burden)	
*Clinicians do not like the ASP because they feel it limits their ability to treat patients the way they think is best (limiting)+	
*Clinicians think the ASP delays antibiotic therapy too much (delay)+~	
*Clinicians think the ASP restricts too many antibiotics (restricting)	
*The ASP involves too many steps for clinicians to adhere to in prescribing antibiotics (excessive)	
Construct 7. Engaging 	
The ASP had the support of the key opinion leaders in the hospital (support)	
The ASP has considerable visibility within the hospital (visibility)	
I work well with the interdisciplinary medical teams (collaborative)>	
I work well with individual clinicians (individual)>	
Note. Paired symbols for items within constructs >^+~ indicate residuals
allowed to correlate.
Item-Item correlations between Engaging and Self Efficiency
	authority	Decision
 making	responsible_for
outcomes	hopeful	skillful	accomplishment	Empowered 
to improve	invested_in 
success	
support	0.25	0.37	0.45	0.53	0.48	0.58	0.5	0.21	
visiability	0.58	0.31	0.53	0.55	0.62	0.65	0.51	0.4	
collaborative	0.41	0.66	0.4	0.38	0.36	0.43	0.27	0.54	
individual	0.37	0.58	0.31	0.42	0.36	0.37	0.27	0.49	
